# BAFF-driven NLRP3 inflammasome activation in B cells

**DOI:** 10.1038/s41419-020-03035-2

**Published:** 2020-10-01

**Authors:** Ken-Hong Lim, Lih-Chyang Chen, Kate Hsu, Chia-Ching Chang, Chia-Yu Chang, Chen-Wei Kao, Yi-Fang Chang, Ming-Chih Chang, Caleb Gonshen Chen

**Affiliations:** 1 Department of Hematology, MacKay Memorial Hospital, Taipei, 10449 Taiwan; 2 Department of Medicine, MacKay Medical College, New Taipei City, 25245 Taiwan; 3 Department of Hematology, GCRC Laboratory, Mackay Memorial Hospital, New Taipei City, 25160 Taiwan; 4 Department of Medical Research, Transfusion Medicine & Immunogenetics Labatory, Mackay Memorial Hospital, New Taipei City, 25160 Taiwan; 5 Department of Nursing, MacKay Junior College of Medicine, Nursing, and Management, New Taipei, Taiwan; 6 College of Biological Science and Technology, National Chiao Tung University, Hsinchu, 300 Taiwan; 7 Center for Intelligent Drug Systems and Smart Bio-devices (IDS2B), National Chiao Tung University, Hsinchu, 300 Taiwan; 8 Department of Electrophysics, National Chiao Tung University, Hsinchu, 300 Taiwan; 9 Institute of Physics, Academia Sinica, Nankang, Taiwan; 10 Institute of Molecular Medicine, National Tsing-Hua University, Hsin-Chu, Taiwan

**Keywords:** Immune cell death, Inflammasome

## Abstract

BAFF supports B-cell survival and homeostasis by activating the NF-κB pathway. While NF-κB is also involved in the priming signal of NLRP3 inflammasome, the role of BAFF in NLRP3 inflammasome regulation is unknown. Here we report BAFF engagement to BAFF receptor elicited both priming and activating signals for NLRP3 inflammasomes in primary B cells and B lymphoma cell lines. This induction of NLRP3 inflammasomes by BAFF led to increased NLRP3 and IL-1β expression, caspase-1 activation, IL-1β secretion, and pyroptosis. Mechanistically, BAFF activated NLRP3 inflammasomes by promoting the association of cIAP-TRAF2 with components of NLRP3 inflammasomes, and by inducing Src activity-dependent ROS production and potassium ion efflux. B-cell receptor (BCR) stimulation on the Lyn signaling pathway inhibited BAFF-induced Src activities and attenuated BAFF-induced NLRP3 inflammasome activation. These findings reveal an additional function of BAFF in B-cell homeostasis that is associated with BCR activities.

## Introduction

B cell-activating factor (BAFF), a member of the tumor necrosis factor (TNF) family, maintains B cell homeostasis^1^. This homeostasis of mature B lymphocytes is known to mediate survival through BAFF receptor 3 (BR3, also known as BAFFR)^2,3^ or through coordinated B-cell receptor (BCR) signaling^4,5^. BAFF is also necessary for maintaining the homeostasis for normal B cell development^6^. Stimulation of BAFFR recruits TNF receptor-associated factor 3 (TRAF3), resulting in further release of NF-κB-inducing kinase (NIK). This strongly activates the alternative nuclear factor-B2 (NF-κB2) pathway and weakly activates the classical NF-κB1 pathway in B cells^1^. Mice treated with reagents that block BAFF binding to BAFFR resulted in loss of most follicular cells, while mice with transgenically induced elevation of BAFF expression showed increased number of B cells and also developed autoimmune pathologies^7,8^. A more recent study found that BAFF binding to BAFFR contributed to early activation of spleen tyrosine kinase (Syk) by activating the Src family of protein kinases (SFKs) and by binding to the phosphorylated immunoreceptor tyrosine-based activation motif (ITAM) of Ig-α^9^. B cells express a number of SFKs including Lyn, Fyn, Blk, Hck, and Fgr^10,11^. Lyn, the predominant SFK in B cells, limits BCR activation by triggering a negative regulatory feedback reaction mediated by phosphatases. By recruiting C-terminal Src tyrosine kinase (CSK) to lipid rafts^10^, activation of Lyn after BCR ligation could inhibit the activity of all SFKs including Lyn itself^11^.

Moreover, NF-κB is critical for regulating inflammatory and immune responses^12^ by signaling the initiation of inflammasome activation^13,14^. By up-regulating the expression and release of proinflammatory cytokines like IL-1β and IL-18, inflammasomes drive innate immune responses^14^. Inflammasomes are multimeric complexes that comprise of an adaptor protein named apoptosis-associated speck-like protein containing a CARD (ASC) and an inactive zymogen, procaspase-1, together with a sensor protein that is either absent in melanoma 2 (AIM2) receptor, AIM2-like receptor (ALR), or one from the NOD-like receptor (NLR) family^15,16^. Priming signals like lipopolysaccharides (LPS) and TNF activate NF-κB transcription factor and potently induce NLR and pro-IL-1β^17,18^. Activation of inflammasome requires a second signal that induces the autocatalytic cleavage of procaspase-1 to caspase-1. This mediates membrane pore formation^19,20^, which in turn controls the release of cellular contents and rupture of cell membrane.

We previously found high levels of serum BAFF and increased B cell activation in patients with essential thrombocythemia (ET)^21,22^. The activated B cells in these ET patients expressed high levels of IL-1β. Interestingly, the B cell counts in these ET patients were significantly lower than in healthy adults. Thus, we hypothesized that B cells could have undergone inflammasome activation and subsequent cell death through activation of the BAFF–BAFFR axis in these ET patients. Here we explored the potential mechanism of activating signals for inflammasome in B cells responding to BAFF.

## Materials and methods

### Cell lines and cell culture

Materials used in this study are listed in Supplementary Information. JM-1 and SU-DHL4 lymphoma cells were obtained from ATCC grown in RPMI 1640 medium (Life Technologies). To enrich primary B cells from healthy donors, B cells were isolated using human CD19-positive selection kit (StemCell Technologies) from peripheral blood mononuclear cells (PBMCs). To prepare immunoblotting, CellXVivo Human B Cell Expansion Kit (not containing BAFF verified by R&D Systems) was used to expand CD19+ isolated B cells. All healthy donors provided informed consent approved by the MacKay Memorial Hospital Institutional Review Board (12MMHIS034, 18MMHIS055) and was carried out in accordance with the principles of the Declaration of Helsinki.

### Quantification of active caspase-1

B cells were plated at 5 × 10^5^ cells/well in 12-well dishes, treated with BAFF or reagents as indicated. Equivalent amount of lysates were assayed for their ability to cleave a fluorescent caspase-1 substrate, YVAD-AFC according to the manufacturer’s protocol (Abcam). Values were normalized to phosphate-buffered saline (PBS) controls. All conditions were run in duplicate wells and three independent experiments were performed for each time point.

### Immunoblotting

Cells were lysed in RIPA buffer, and whole-cell extracts were quantified by the Bradford assay (Bio-Rad). For assessment of IL-1β secretion and caspase-1 release, culture supernatants were collected, mixed with a 1/10 volume of 100% (wt/vol) trichloroacetic acid, and incubated for 10 min at 4 °C. The precipitated protein samples or cell lysates were resolved by SDS/PAGE and transferred to PVDF membranes (Millipore). The membranes were then incubated with the indicated primary antibodies, followed by an HRP-conjugated secondary antibody. The immunoreactive bands were detected using the Western Lighting Plus-ECL system (PerkinElmer) or the Opti-ECL HRP reagent kit (BIOMAN).

### Flow cytometric staining and analysis

B cells were stained with fluorescent-labeled antibodies and fixed with 4% paraformaldehyde and examined by FACScalibur (BD). Caspase-1 activity of B-cell populations was determined by FACS after labeling with the fluorogenic substrate FAM-YVAD-FMK (FLICA) for 30 min. For cell death determination, cells were stained with Annexin-V conjugated with FITC or APC combined with propidium iodide (PI). To measure ROS, B cells were incubated with chloromethyl-H2-2′,7′-dichlorodihydrofluorescein diacetate (DCF, Invitrogen) for 20 min. N-acetyl-l-cysteine (NAC) as a scavenger of ROS, (2*R*,4*R*)-4-aminopyrrolidine-2,4-dicarboxylate (APDC) as a ROS inhibitor, and diphenyleneiodonium (DPI) as a NAPDH oxidase inhibitor were used to explore the ROS examined by FACScalibur. Data were analyzed using Cell Quest Pro software (BD).

### Immunohistochemistry and NLRP3 and ASC speck detection

B cells were pretreated with zVAD-FMK for 30-min and then treated with BAFF for 2-h. Cells were blocked using 1% BSA followed by incubation with anti-NLRP3 or anti-ASC antibodies (1:1000). The slides were washed with PBS and mounted using ProLong Gold mounting medium containing DAPI (Invitrogen). The data were expressed as the percentage of NLRP3 and ASC specks per number of cells per field.

### Measurement of K^+^ efflux

Ten million B cells were treated with BAFF 1-h in the absence or presence with PP1 30-min prior to BAFF exposure. After 1-h, the extracellular medium was removed and centrifuged at 10,000 × *g* 15-s to pellet cells. One hundred microliter of 65% nitric acid was used to resuspend the cell pellet and this was stayed at 60 °C 3-h to ensure cell rupture and bring the cell suspension to a total volume of 5 mL by adding the distilled water. Liquid chromatography–mass spectrometry experiments were performed using an Impact HD Q-TOF mass spectrometer (Bruker, Germany), which was equipped with an electrospray ionization (ESI) source operating in positive ion mode.

### Statistical analysis

To compare means between two independent groups that were not normally distributed, the nonparametric Mann–Whitney *U* test was used. If two groups were normally distributed, Student’s *t*-tests were applied to test for differences. To compare the change of caspase-1 activity of human primary B cells after treatment with BAFF, paired *t*-tests were used. All data are typically presented as a pool of three experiments (mean ± s.e.m.). The threshold for statistical significance was defined at *p* < 0.05. GraphPad Prism 6 (GraphPad Software) or SPSS 12.0 (SPSS Inc., Chicago, IL, USA) were used for all analyses.

## Results

### BAFF-induced NLRP3 inflammasome activities

**Fig. 1:**
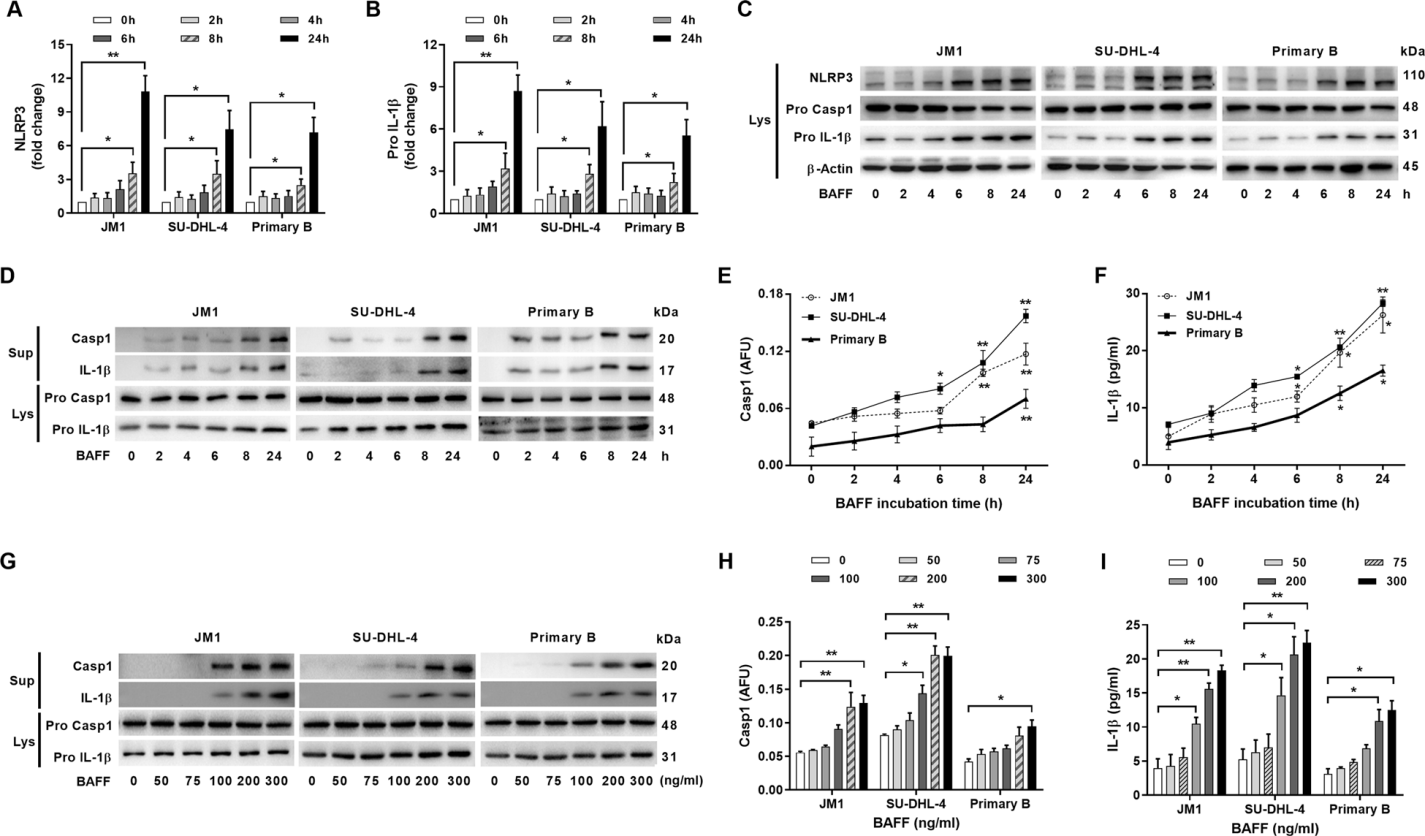
NLRP3 inflammasome expression and activity levels in B cells were responsive to BAFF stimulation in a time-dependent and dose-dependent manner. **a**–**c** The levels of NLRP3 **a** and pro-IL-1β **b** in B cells were determined using quantitative RT-PCR after the treatment with BAFF (200 ng/ml) over time. The levels of mRNA (fold change) in treated cells were compared to that of the untreated cells. Primary B cells were isolated using CD19 MACS beads prior to incubation with BAFF. Caspase-1 activity and IL-1β of CD19+ isolated B cells from PBMC were determined (Fig. S2). **c** Western blots showed the expression levels of NLRP3 and its targets at the protein levels. **d**–**f** BAFF-stimulated processing of pro-caspase-1 and pro-IL-1β. **d** Immunoblot analyses of mature caspase-1 and IL-1β molecules in cell lysates and culture supernatants. JM1, SU-DHL-4, and primary B cells were left untreated or treated with BAFF (200 ng/ml) for the indicated length of time. **e** The caspase-1 activities in treated lymphoma or primary B cells were quantified by fam-FLICA fluorescence spectrometry, and **f** IL-1β released in culture supernatants was measured by ELISA. AFU, arbitrary fluorescence units. **g** The lysates and culture supernatants of B cells treated with BAFF for 24-h at concentrations ranging from 50 to 300 ng/ml were analyzed by immunoblotting for caspase-1 cleavage and concurrent IL-1β maturation. **h** The caspase-1 activities in the treated B cells and IL-1β released in culture supernatants **i** were measured using fam-FLICA fluorescence spectrometry and IL-1β ELISA, respectively. Asterisks represent significant differences between BAFF stimuli and the untreated baseline. These cell-based studies were performed at least three times and showed comparable results. *p < 0.05, **p < 0.01 (Student’s *t*-test). The results were presented as mean ± SEM. Lys lysate, Sup supernatants, PB primary B cell.

We first investigated whether BAFF could modulate the expression of *NLRP3* and *pro-IL-1β* in B cells. Using real-time PCR, we measured mRNA levels for *pro-IL-1β*, *NLRP3*, *NLRP1*, and *NLRC4* in response to BAFF stimulation. In contrast to NLRP3 and pro-IL-1β whose expression levels were significantly up-regulated by BAFF in the three types of B cells tested, the levels of NLRP1 or NLRC4 did not increase by BAFF (Figs. 1a, b and S1). Significant increase in the protein expression of NLRP3 and pro-IL-1β was also noted after 8-h treatment with BAFF (Fig. 1c).

To test whether BAFF activated through proteolytic processing of pro-caspase-1 and pro-IL-1β, we treated two lymphoma cell lines and primary B cells with different concentrations of BAFF. Immunoblotting analyses of cleaved caspase-1 (p20) and IL-1β (p17) showed that their cleavage processing started remarkably within 8-h after addition of BAFF, and increased further over time (Fig. 1d). Consistently, the levels of active caspase-1 (Fig. 1e) and IL-1β (Fig. 1f) increased post-BAFF treatment.

We next explored the effects of different concentrations of BAFF on inflammasome activities of B cells. Pro-caspase-1 cleavage was determined by measuring the production of the p20 subunit, and concurrent processing of pro-IL-1β was by measuring its mature p17 fragment. Increasing concentrations of BAFF in the treatments enhanced production of cleaved caspase-1 and mature IL-1β (Fig. 1g–i). These findings indicate BAFF plays a role in modulating NLRP3 inflammasome expression and activation in a time-dependent and dose-dependent manner.

### The cIAP–TRAF2 complex promoted BAFF-mediated caspase-1 processing

**Fig. 2:**
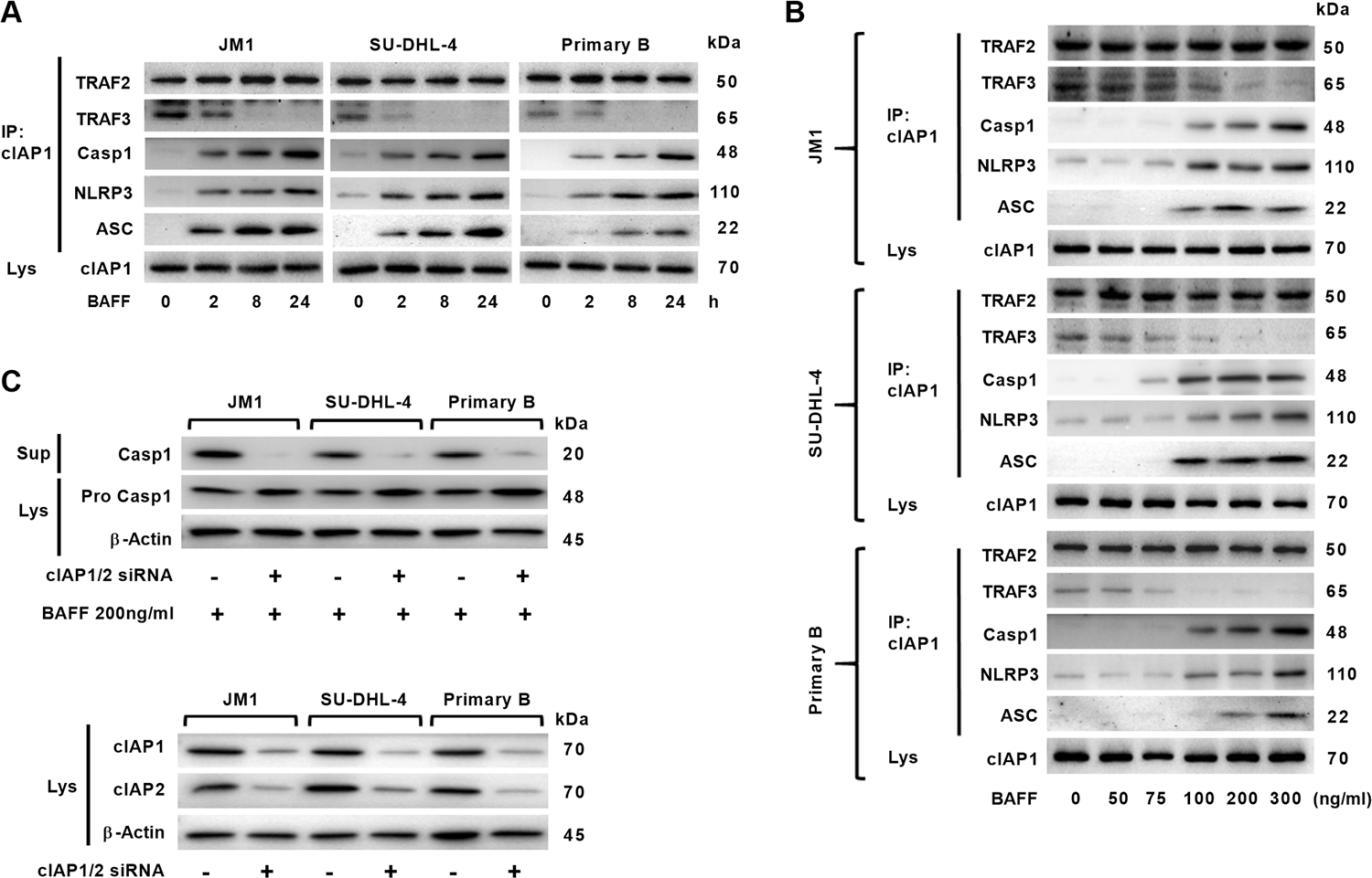
Dynamic changes of the expression of NLRP3 inflammasome components were inversely proportional to TRAF3 expression. **a** JM1, SU-DHL-4, and primary B cells were treated with BAFF (200 ng/ml) for the indicated periods of time. cIAP1 in cell lysates was immunoprecipitated with cIAP1 antibody and protein G beads. Co-precipitated TRAF2, TRAF3, pro-caspase-1, NLRP3, and ASC proteins were detected by immunoblot. **b** Lymphoma or primary B cells were treated with BAFF at various concentrations for 2-h. The co-precipitated proteins with cIAP1 were analyzed as in **a** by Western blot. **c** B cells were transfected using lipofectamine with scrambled siRNA as controls (indicated by −) and siRNA that targets cIAP1 (0.5 nM) and cIAP2 (0.5 nM), prior to BAFF treatments for 24-h. Caspase-1 cleavage was analyzed by Western blot. Cell-based studies were performed at least three times, and showed similar findings. The knockdown effects of cIAP1/2 siRNA were analyzed using real-time PCR at 24-h post-transfection (data not shown).

When BAFF binds to BAFFR, TRAF3 is recruited to the receptor to be degraded, which promotes NF-κB activation and leaves cIAP–TRAF2 in the cytoplasm^1^. As the cIAPs–TRAF2 complex is known to mediate caspase-1 activation^23^, we investigated any possible interaction between the cIAP–TRAF2 and the NLRP3–ASC–procaspase-1 complexes. By co-immunoprecipitation experiments in B cells, we found pro-caspase-1, NLRP3, and ASC co-precipitated with cIAP1–TRAF2. In contrast, the levels of TRAF3 decreased following BAFF treatments (Fig. 2a, b). These phenomena were replicated in all three types of B cells. To test whether cIAPs could mediate pro-caspase-1 processing, we employed RNAi to knock down expressions of cIAP1 and cIAP2 in B cells. When cIAP1/2 expressions were silenced in B cells prior to 24-h BAFF incubation, the amount of cleaved caspase-1 decreased markedly compared to the controls without silencing of cIAP1/2 (Fig. 2c). These results indicate cIAP–TRAF2 was associated with pro-caspase-1-containing complexes in B cells following BAFF treatments in a dose-dependent and time-dependent fashion. In addition, cIAPs enable caspase-1 catalysis in B cells stimulated with BAFF.

### BAFF activated the assembly of NLRP3–ASC–procaspase-1 complexes

**Fig. 3:**
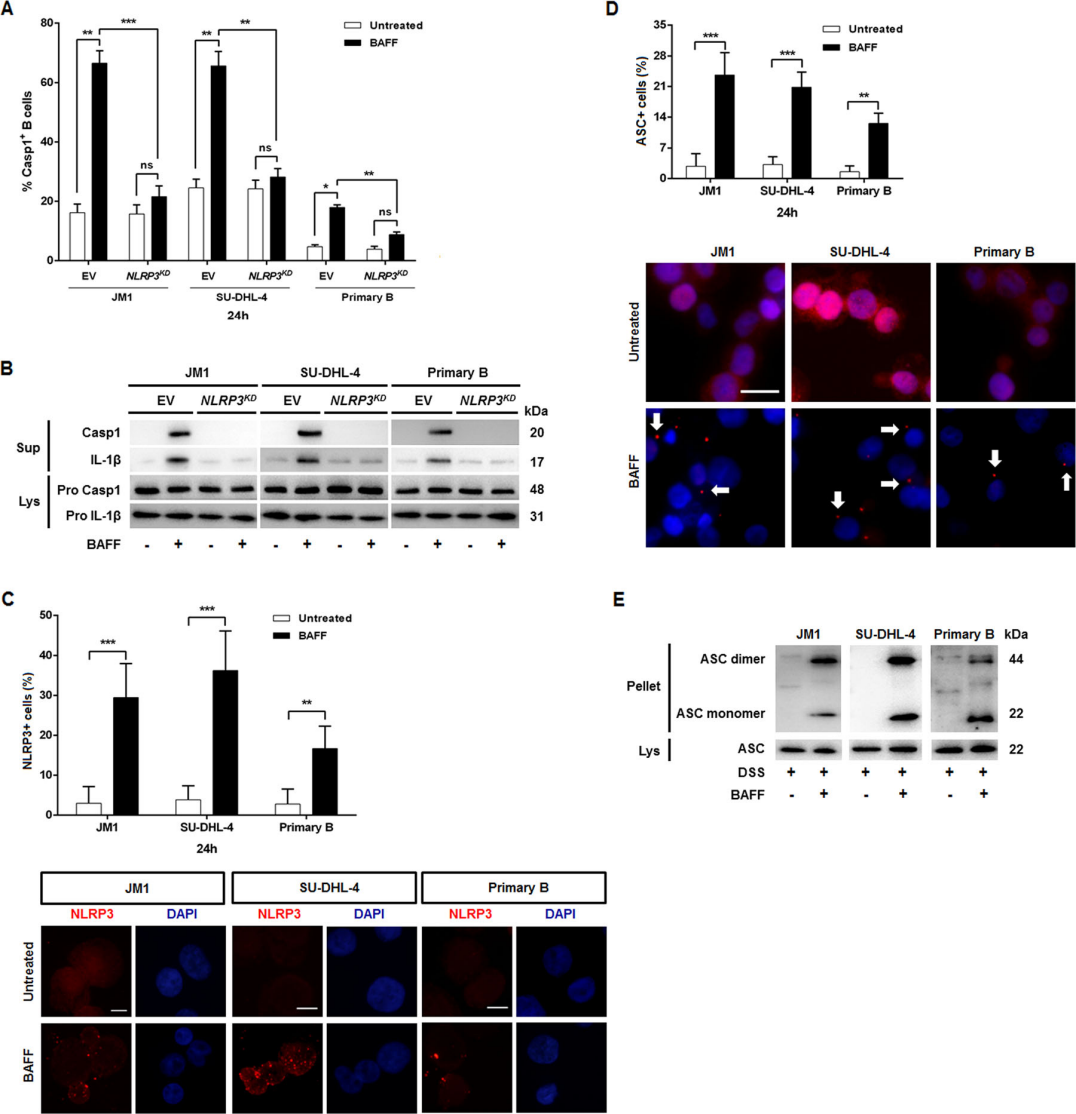
BAFF-mediated NLRP3 inflammasome activities. NLRP3 knockdown (KD) lymphoma cells were established by transfection with a lentiviral vector encoding NLRP3 shRNA or with an empty vector (EV) as the control. For primary B cells, NLRP3 knockdown were accomplished using its specific siRNA. Mouse anti-NLRP3 was used to evaluate the KD effects (data not shown). **a** Using a fluorescent inhibitor of active caspase-1 (fam-FLICA), BAFF treatments were shown to activate caspase-1 in B cells. **b** Cell lysates from the treatment in **a** were analyzed by immunoblot for caspase-1 cleavage and for the mature IL-1β fragment. **c** The percentages of NLRP3 speck-positive cells in BAFF-treated and untreated cells were determined. Representative immunofluorescence images (×100 magnification) of JM1, SU-DHL4, and primary B cells pre-incubated with pan-caspase inhibitor zVAD-FMK stained with anti-NLRP3 antibody (red) and DAPI (blue) with or without BAFF treatments were shown. Scale bar, 10 μm. **d** Cells were observed and photographed by fluorescence microscopy (×40 magnification). Images were taken with a BX40 imaging upright microscope (Olympus), and captured using an AxioCam HRm camera (Olympus) with AxioVision Software. Untreated controls did not form ASC specks, while BAFF-treated cells formed large perinuclear ruby fluorescent ASC specks. The percentages of cells with ASC specks were calculated by dividing the number of cells with ASC specks over the total number of counted cells. The cell-based studies were performed at least three times with comparable results. Scale bar, 25 μm. To quantify the speck formation, the percentage of cells that contained an ASC speck was determined. Cells from 10 different fields (average of 250 cells/field) were counted. Images were analyzed using ImageJ (rsb.info.nih.gov). **e** B cells were treated with BAFF (200 ng/ml) or not for 2-h in the presence of zVAD-FMK, and then lysed. The ASC proteins present in the lysates were pelleted by centrifugation at 5000 rpm as described under ‘Materials and methods’ section. The pellets (Pell) was incubated with DSS for 30 min. The lysates (Lys) were not treated with DSS. The Lys and Pell were then fractionated by SDS–PAGE and Western blotted with anti-ASC antibodies. *p < 0.05, **p < 0.01, ***p < 0.001 (Student’s *t*-test). Results are represented as mean ± SEM.

To validate the effects of NLRP3–ASC–procaspase-1 complex in BAFF-directing activation of inflammasomes, we measured inflammasome activities in *NLRP3*-deficient B cells incubated with BAFF. *NLRP3*-knockdown in the three types of B cells abolished the effects of BAFF on promoting caspase-1 activity and IL-1β secretion (Fig. 3a). Supporting these observations, immunoblotting analyses showed that BAFF-potentiated processing of pro-caspase-1 and pro-IL-1β was substantially impaired in NLRP3^KD^ B cells (Fig. 3b). Inflammasomes are multi-protein complexes that minimally consist of NLR protein, ASC, and caspase-1^14^. To assess the degree of NLRP3 oligomerization in B cells stimulated by BAFF, we performed immunostaining for NLRP3 in combination with DAPI. BAFF-treated JM1, SU-DHL4, and primary B cells consistently showed that NLRP3 aggregated in cytoplasm, as shown by fluorescence microscopy (Fig. 3c). We next examined inflammasome complex assembly after activating signals by measuring ASC oligomerization in B cells treated with BAFF^24^ (Fig. 3d). Cell-free pellets were then chemically cross-linked to non-cleavable proteins using DSS to determine the oligomeric state of ASC in the cells. BAFF-treated cells yielded cross-linked oligomers, while untreated controls did not (Fig. 3e). These data suggest BAFF can trigger NLRP3 inflammasome activation in B cells.

### ROS production and K^+^ efflux were two contributors to BAFF-mediated NLRP3 activation

**Fig. 4:**
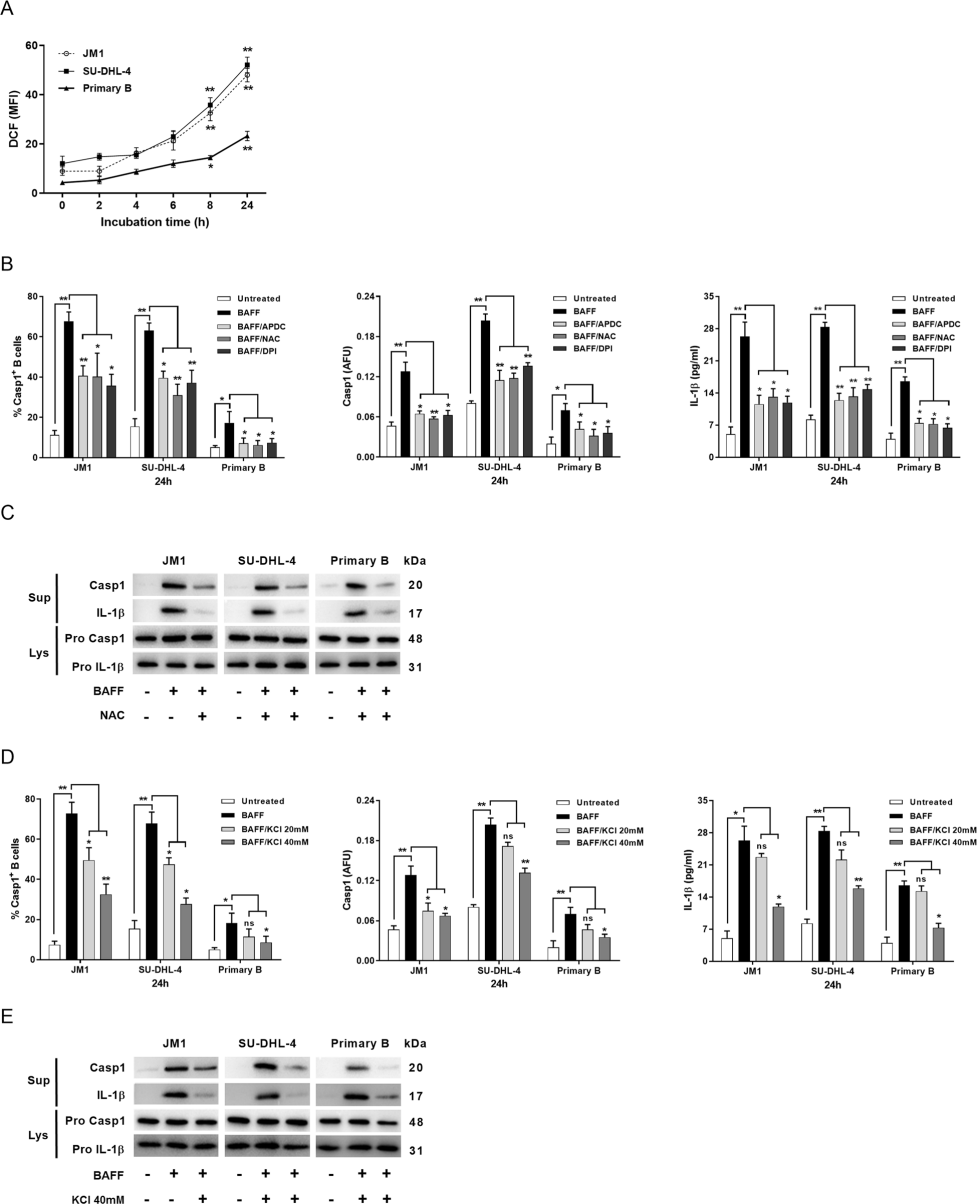
BAFF activation of inflammasome signaling was dependent on ROS production and potassium efflux. **a** JM1, SU-DHL-4, and primary B cells were treated with BAFF for the indicated periods of time, and ROS production was analyzed using the fluoroprobe DCF. Experiments were repeated three times. **b** B cells were stimulated with BAFF in the absence or presence of ROS inhibitors APDC (50 μM) and NAC (25 mM) or NOX inhibitor DPI (25 μM). Caspase-1-activated fractions were determined using flow cytometric analysis (left panel) and caspase-1 activities quantified by fam-FLICA fluorescence spectrometry (middle panel) after 24-h incubation. After priming with BAFF for 24-h in the absence or presence of the ROS or NOX inhibitors, the whole-cell lysates of B cells were collected for in vitro caspase-1 assay. Supernatants from the treated B cells were collected for IL-1β ELISA (right panel). **c** Representative immunoblots of caspase-1 and IL-1β of BAFF-treated B-cell lysates and supernatants in the absence or presence of NAC were shown. **d** B cells were treated with BAFF in the absence or presence of KCl at 20 or 40 mM. The percentages of B cells expressing active caspase-1, and the levels of active caspase-1 and IL-1β were determined for JM1, SU-DHL-4, and primary B cells. **e** Immunoblotting analyses of caspase-1 (p20) and IL-1β (p17) were conducted for BAFF-treated B cells, with or without co-treatments of 40 mM KCl **a**, **b**, **d**. The data were from three independent experiments. **a**, **e** One of the two independent experiments was shown. Asterisks indicate significant differences due to the indicated treatments. *p < 0.05; **p < 0.01 (two-tailed, unpaired Student’s *t*-test); ns non-significant. The results were presented as mean ± SEM. AFU arbitrary fluorescence units.

The cleaved forms of caspase-1 and IL-1β appeared in cell supernatants within 2-h after BAFF treatment (Fig. 1d). However, pro-caspase-1 binding to cIAP1–TRAF2 complex was observed after 2-h of BAFF treatment (Fig. 2a). We speculated that other molecular mechanisms were intertwined in inflammasome activation. We examined the dependence of NLRP3 activation by BAFF on intracellular ROS and K^+^ efflux. Intracellular ROS is essential for inflammasome activation^25,26^, and K^+^ efflux is regarded as the most common mediator of NLRP3 activation in response to diverse stimulators^27^. The intensities of DCF corresponds to the content of ROS, and increased after 6-h of incubation with BAFF and the increase persisted over time in all three types of B cells (Fig. 4a). ROS inhibitors, APDC and NAC, and NADPH oxidase (NOX) inhibitor DPI dampened caspase-1 activity and reduced the amount of IL-1β in supernatants, as assessed by fluorescence spectrometry and ELISA, respectively (Fig. 4b). Immunoblotting revealed that BAFF-activated cleavage of pro-caspase-1 and pro-IL-1β was also reduced by NAC (Fig. 4c), agreeing with the fluorescence spectrometric and ELISA findings (Fig. 4b).

By subjecting the cells to high extracellular KCl and inhibiting K^+^ efflux, BAFF-induced caspase-1 activity and IL-1β production in B cells were markedly attenuated (Fig. 4d). These observations are consistent with the findings from the immunoblots of the supernatants and lysates of B cells treated with high KCl (Fig. 4e).

Cell-surface P2X_7_ receptor (P2X_7_R) is involved in ATP-induced intracellular K^+^ efflux, NLRP3 inflammasome activation, and IL-1β secretion^27,28^. ATP-induced P2X_7_R activation triggers substantial increase in intracellular [Ca^2+^], reflecting P2X_7_R channel opening^28,29^. To examine the ionotropic function of P2X_7_R, B cells were treated with BAFF and loaded with Fluo-4-AM Ca^2+^ indicator dye. Stimulation with BAFF yielded a rapid (within 90 s) and sustained (over 3 min) rise in intracellular [Ca^2+^], while this effect was inhibited by oxidized ATP (Fig. S3). These findings further support that ROS and K^+^ efflux were involved in BAFF-activated NLRP3 inflammasomes in B cells.

### BAFF-activated inflammasomes in B cells through BAFFR

**Fig. 5:**
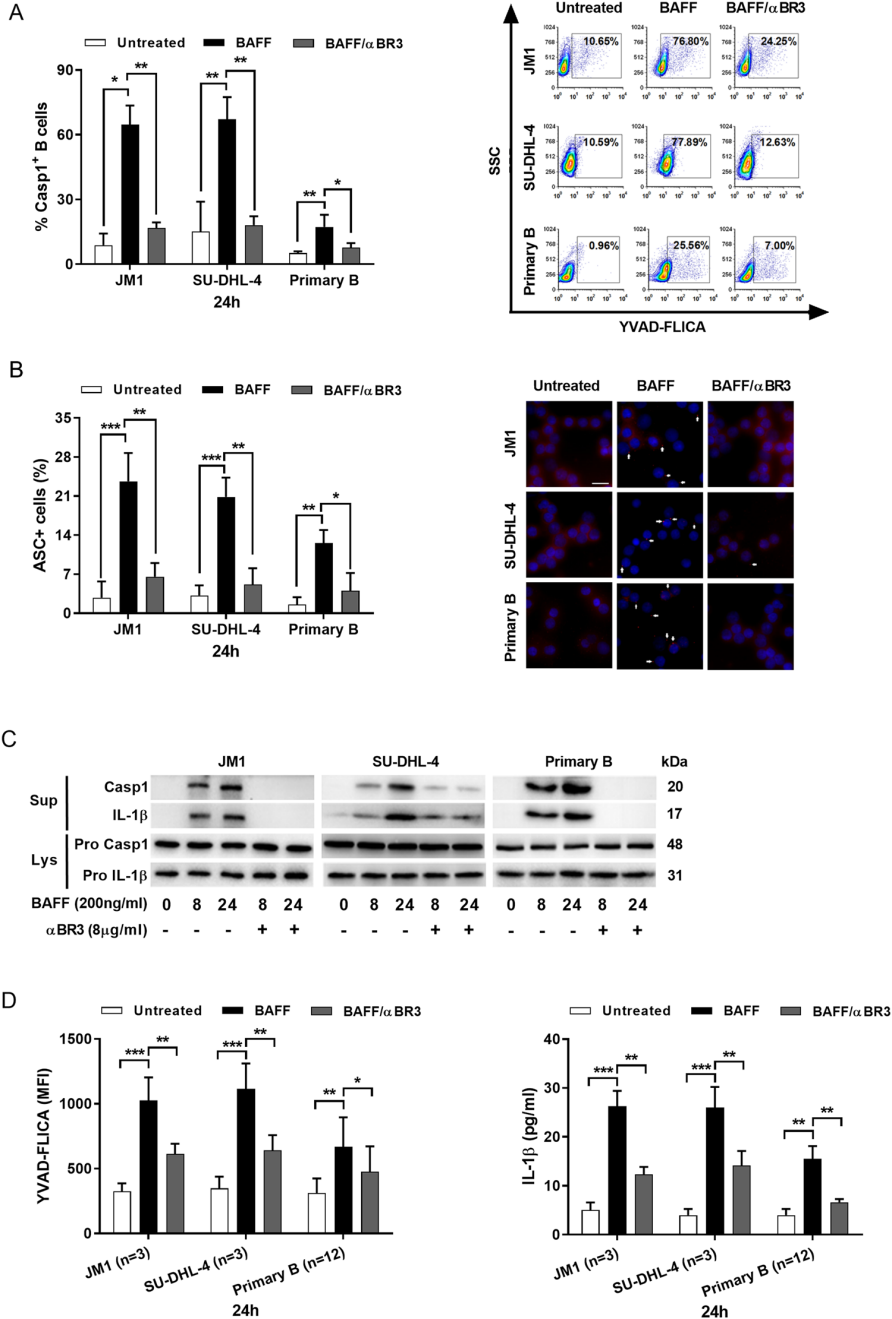
Pro-inflammatory stimulation of BAFF-induced generation of caspase-1 and IL-1β in B cells through BAFFR. **a** B cells were incubated with anti-BR3 neutralizing antibodies (8 μg/ml) for 30 min, followed by 24-h BAFF treatments. The percentages of B cells with active caspase-1 was determined by FLICA flow cytometry (left panel). The results were presented as mean ± SEM (*n* = 3); the representative dot plots of FLICA staining were shown (right panel). **b** Speck-forming cells were analyzed by fluorescence microscopy (×40 magnification). The number of specks was quantified and expressed as the percentage of the number of cells (scale bar, 25 μm; left panel). Representative photographs of immunofluorescence counterstaining for ASC (red) and nuclei (blue) for each treatment presented. **c** Immunoblot analyses of caspase-1 and IL-1β processing. JM1, SU-DHL-4, and primary B cells were left untreated or treated with BAFF (200 ng/ml) in the absence or presence of anti-BAFFR antibodies for the indicated length of time. The data presented were from three independent experiments. **d** The mean fluorescent intensity (MFI) of FLICA (left panel) was measured in B cell lines and isolated B cells from healthy donors (*n* = 12) after 24-h BAFF treatments. The levels of IL-1β in cell supernatants was measured by ELISA (right panel). *p < 0.05, **p < 0.01, ***p < 0.001 (Student’s *t*-test). Results were presented as mean ± SEM.

BAFF transduces signals in B cell receptors including BAFFR, transmembrane activator and calcium modulator and cyclophilin ligand interactor (TACI), and B cell maturation antigen (BCMA)^1^. We next investigated the receptors involved in BAFF-elicited caspase-1 activity. After blocking the receptors with their corresponding antibodies, caspase-1-activated B cell populations were measured by FLICA assay. We found that anti-BAFFR antibodies impaired BAFF-induced caspase-1 activity in B cells (Figs. 5a and S4). To provide additional evidence that caspase-1 is activated in axis signaling by forming the supramolecular assembly of ASC, we performed bioimaging analyses on BAFF-treated B cells in the absence or presence of anti-BAFFR antibodies. ASC speck formation was significantly reduced after blocking BAFFR in all three types of B cells (Fig. 5b). Similarly, anti-BAFFR antibodies significantly impaired processing of pro-caspase-1 and pro-IL-1β precursors (Fig. 5c). To determine caspase-1 activity and IL-1β release, we used flow cytometer to assess the fluorescence intensity of FLICA that reacts with caspase-1, and performed ELISA to measure secreted IL-1β. After blocking the BAFF–BAFFR axis, BAFF-induced caspase-1 activity and IL-1β release were drastically limited (Fig. 5d). These findings suggest that BAFF-stimulated NLRP3 inflammasome activation in B cells required BAFFR.

### BAFFR transmitted signals through Src-family kinase

**Fig. 6:**
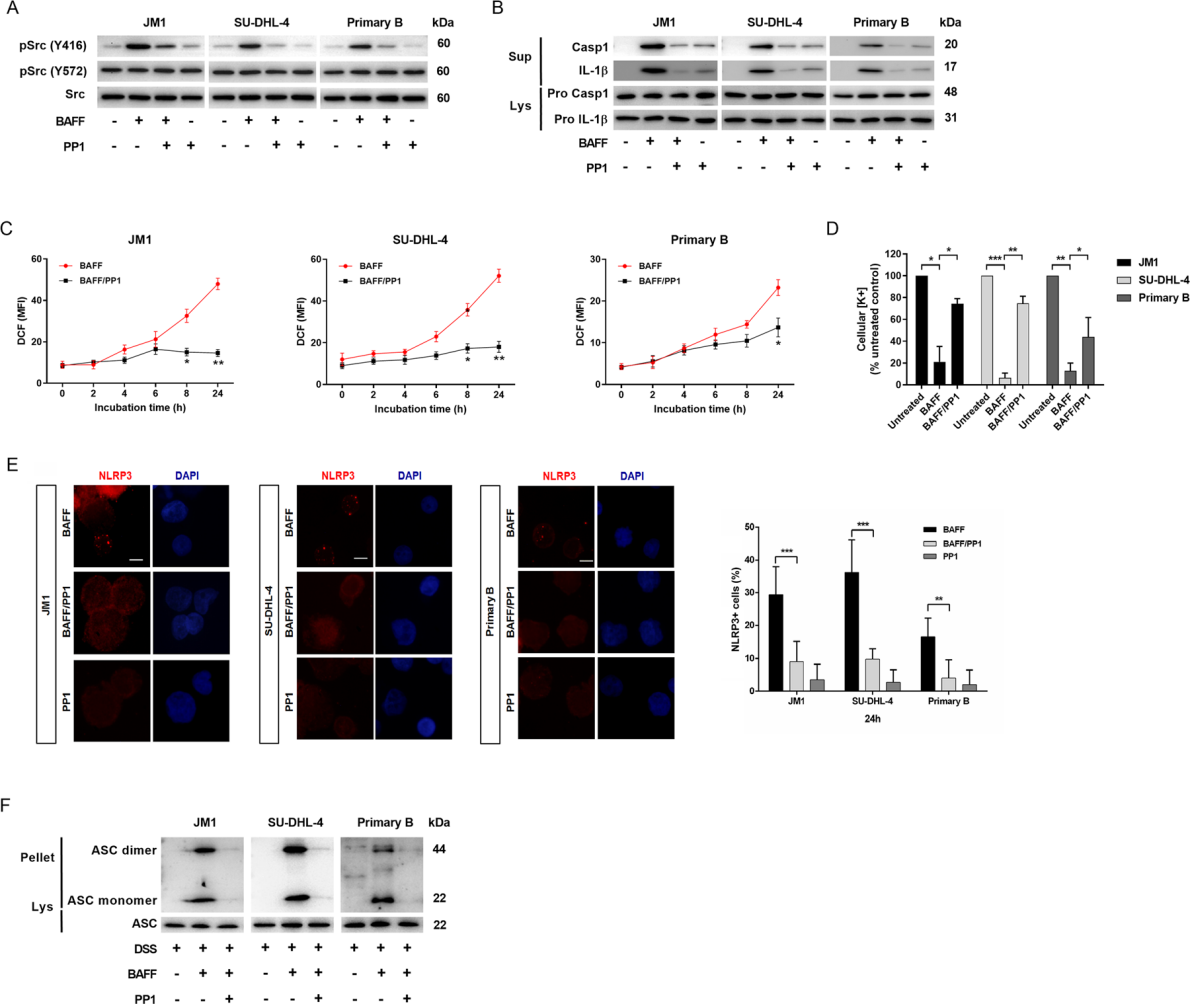
BAFF-induced ROS production, K+ efflux, and NLRP3 inflammasome assembly by activation of Src kinase. **a** Activity of key signaling components of the pathways was assessed using phospho-specific antibodies against SRC(Y416) and SRC(Y527) in B cells treated with BAFF for 30 min. Western Blots were then stripped and reprobed with antibodies against total-SRC. **b** B cells were treated with PP1 (10 μM) for 30 min followed by BAFF stimulation for 24-h. Their lysates and culture supernatants were analyzed by immunoblot for caspase-1 cleavage and concurrent IL-1β maturation. **c** ROS production was determined using fluoroprobe DCF. JM1, SU-DHL-4, and primary B cells were treated with BAFF in the absence or presence of PP1. **d** B cells treated with PP1 for 30 min prior to 1-h BAFF incubation were lysed with 30% nitric acid and the lysates were analyzed by mass spectrometer to measure the concentration of cellular potassium ions. **e** The representative immunofluorescence images (×100 magnification) were on BAFF-treated JM1, SU-DHL4, and primary B cells stained with anti-NLRP3 antibody (red) and DAPI (blue). Scale bar, 10 μm. **f** B cells were left untreated or treated with PP1 for 30 min, followed by BAFF (200 ng/ml) stimulation for 2-h in the presence of zVAD-FMK and subsequent lysis. The ASC proteins present in the lysates were pelleted by centrifugation. The pellets were incubated with DSS for 30 min, while the lysates were left untreated with DSS. The Pell and Lys were then fractionated by SDS–PAGE and analyzed by Western blot with anti-ASC antibodies. Lys lysate, Pell pellets. *p < 0.05, **p < 0.01, ***p < 0.001 (two-tailed, unpaired Student’s *t*-test). As a control for the pharmacological inhibitors, the equivalent volume of dimethyl sulfoxide (DMSO) was used.

SFKs play a pivotal role in NLRP3 inflammasome activation in response to innate immune activity^30,31^, and they have been identified as transducers of the BAFF–BAFFR signals^9^. Phosphorylation of c-Src at Y416 enhances the kinase activity by stabilizing the activation loop for substrate binding, while phosphorylation at Y527 suppresses the kinase activity towards substrate binding^32^. Phospho-Y416 Src (pSrc-Y416) levels increased in 30-min following stimulation with BAFF, while phospho-Y527 (pSrc-Y527) and total Src levels remained largely unchanged (Fig. 6a). These findings suggest that BAFF induced localized activation of Src but did not activate the entire intracellular Src pool. PP1, a specific SFK inhibitor, significantly decreased release of the cleaved forms of caspase-1 and IL-1β in the supernatants of BAFF-treated B cells (Fig. 6b). We also found that Src inhibitor substantially reduced ROS generation and K^+^ depletion (Fig. 6c, d). From this finding, Src kinase likely mediated ROS production and K^+^ efflux for inflammasome complex formation. To test whether that NLRP3 oligomerization could also be affected by Src kinase, we performed immunostaining for NLRP3 with phycoerythrin-conjugated antibodies in the absence or the presence of PP1, followed by BAFF treatments. NLRP3 aggregates were formed in B cells after BAFF treatments, and this was abolished by PP1 (Fig. 6e). We also analyzed images of the concurrent FLICA reaction and NLRP3 specks for additional evidence of the caspase-1 activity (Fig. S5). In line with this observation, PP1 abolished ASC oligomerization (Fig. 6f). These data that demonstrate Src kinase could be a potent mediator of the NLRP3 inflammasome assembly upon stimulation by BAFF.

### BCR crosslinking counteracted BAFF-induced inflammasome activation and cell death

**Fig. 7:**
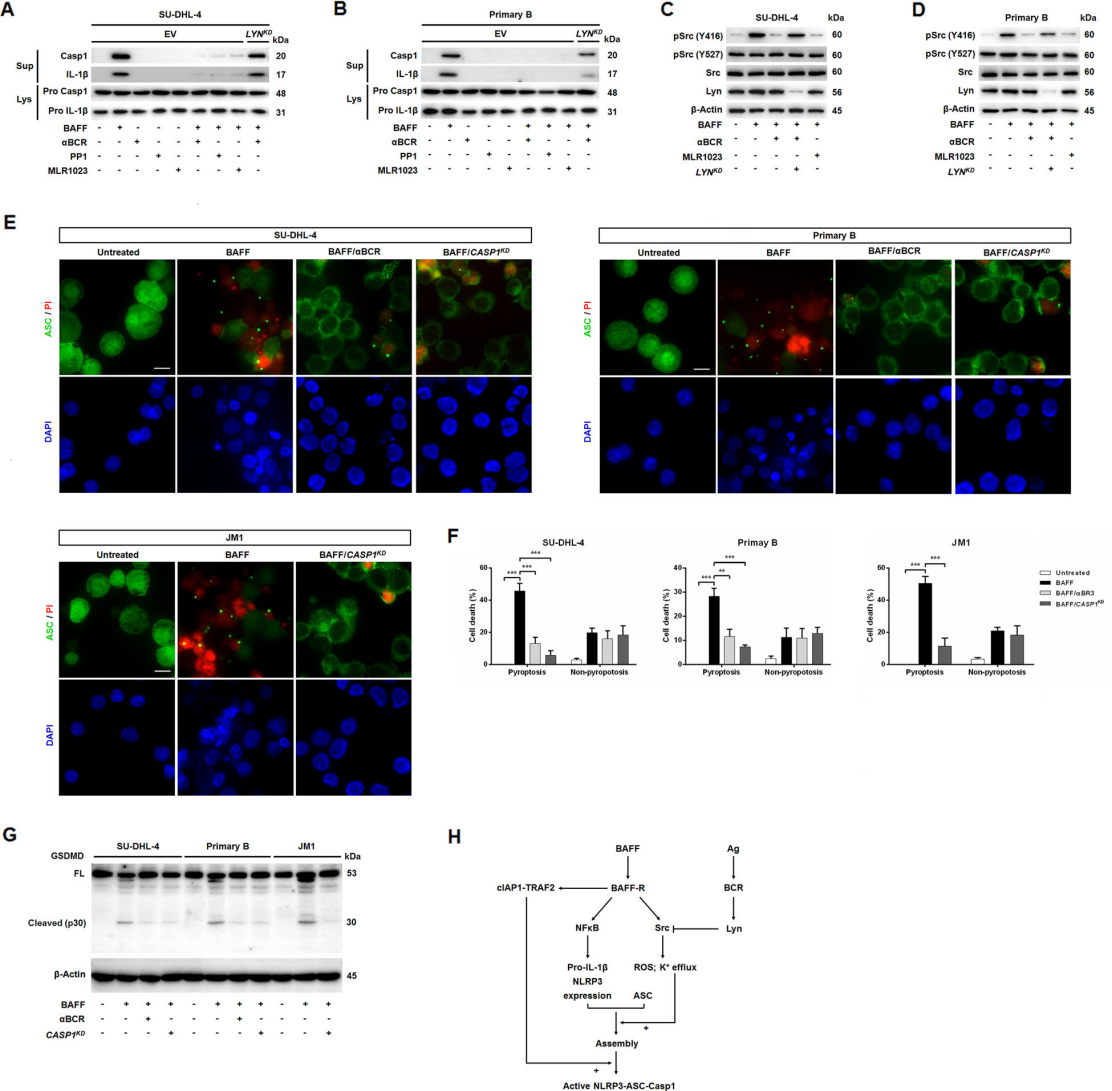
BCR engagement and subsequent Lyn kinase activities counteracted BAFF-induced inflammasome activities and cell death. **a** Maturation of caspase-1 and IL-1β was induced by BAFF, which was then attenuated by pre-incubation with anti-BCR antibodies (1 μg/ml; IgG for SU-DHL-4 cells, IgD + IgM for primary B cells), PP1 (10 μM), and/or MLR1023 (10 μM), in SU-DHL-4 cells transduced with empty vector (EV), comparing to that in stable *LYN*-KD cells. **b** Primary circulating B cells underwent the same treatments as in **a**, except that *LYN* expression was silenced using its siRNA. **c** The activities of the key signaling components in the BAFF–BAFFR axis was assessed using phospho-specific antibodies against SRC (Y416) and SRC(Y527) in BAFF-treated B cells. Blots were then stripped and reprobed with antibodies against total-SRC. Parental SU-DHL-4 and *LYN*-KD SU-DHL-4 cells were treated with BAFF alone or were pre-incubated with anti-BCR antibodies or MLR1023. **d** Primary B cells underwent treatments as in **c**. No experiments were performed in JM1 cells as they lack expression of mature BCR. **e** The representative images of untreated, BAFF-treated, BAFF/anti-BCR-treated, or BAFF/CASP1-KD-treated B cells stained for ASC specks/PI and DAPI were shown (×40 magnification). Scale bar, 30 μm. **f** The percentages of speck-positive and PI-positive cells. For each sample, 500 cells were counted, and ~20 images were analyzed. The data were calculated from three experiments. **p < 0.01, ***p < 0.001 (Student’s *t*-test). The results were presented as mean ± SEM. **g** Western blot analyses of full-length (FL) and cleaved GSDMD (p30) of cell lysates. Parental cells or *CASP1*-KD cells were left untreated or treated with BAFF with BAFF for 8-h in the absence or presence with anti-BCR antibodies. **h** A proposed model of NLRP3 inflammasome activation triggered by BAFF. As a control for the pharmacological inhibitors and activator, the equivalent volume of dimethyl sulfoxide (DMSO) was used. Rabbit anti-caspase-1, and anti-LYN antibodies were used to evaluate the KD effects (data not shown).

The interplay between BCR and BAFF signals is required for B cell survival^4,5,9^. BCR binding to antigen leads to phosphorylation of ITAM in the BCR-associated Igα-chain and Igβ-chain. This initial ITAM phosphorylation is mediated by SFKs including Lyn^33^. Lyn also negatively regulates BCR signaling by inhibiting the activity of all SFK and by recruiting Csk^11,34^. We next examined whether BCR engagement with downstream Lyn kinase could counteract BAFF-induced inflammasome activity in B cells. B cells treated with BAFF in conjunction with anti-BCR antibodies, PP1, or MLR1023 (a specific allosteric activator of Lyn kinase^35,36^), reduced maturation of caspase-1 and IL-1β (Fig. 7a, b). Conversely, these phenomena were not observed in *LYN*-knocked down (KD) B cells. BCR engagement and Lyn kinase activation similarly attenuated BAFF-modulated phosphorylation of Src at Y416 (Fig. 7c, d). Because JM1 cells do not express mature BCR, we omitted the BCR stimulation experiments using JM1 cells.

Caspase-1 also induces rapid lytic cell death termed regulated necrosis or ‘pyroptosis’, which is morphologically different from apoptosis^37,38^. Pyroptosis involves pore formation, osmotic swelling, and early loss of membrane integrity, and is therefore an inflammatory process^39^. Based on these reports, we investigated whether B cells could undergo pyroptosis after the treatment with BAFF. We treated B cells with BAFF for 8-h, and analyzed formation of ASC aggregates with PI staining to determine cell viability (Fig. 7e, f). Pyroptotic cells were positive for ASC specks and PI labeling. BAFF-driven B cell pyroptosis was much dependent on caspase-1 expression, and conversely, this diminished with *CASP1* knockdown. By activating BCR through anti-BCR antibodies, BAFF-induced pyroptosis of B cells was markedly blunted (Fig. 7e). Given the biochemical hallmark of inflammasome-induced pyroptosis is the gasdermin D (GSDMD) undergoing proteolytic process, pore formation generating from N-terminal fragment p30 of GSDMD^19,20^. We performed western blot analyses of full-length and cleaved GSDMD of cell lysates from parental cells, cells pre-incubated with anti-BCR, and *CASP1*-KD cells treated with BAFF or left untreated (Fig. 7g). Indeed, BAFF treatment led to GSDMD cleavage, which was blunted by anti-BCR antibody and CASP1 knockdown.

Altogether, these findings showed that inflammasome activation was stimulated by BAFF engagement, which was associated with Src kinase activity. BAFF-directed inflammasome activities and cell death could be counteracted by BCR ligation and Lyn kinase stimulation, both of which impair Src kinase function.

## Discussion

Inflammasome activation typically requires a priming signal from the NF-κB pathway^14^ and a second signal that triggers subcellular events, such as ROS production^30,40^ and P2X_7_R activation for K^+^ efflux^27,40,41^. Src kinase mediates the second signal of inflammasome to trigger generation of ROS and K^+^ efflux^41,42^. In the present study, we provided evidence that BAFFR interacted with BCR signals to modulate inflammasome activities in B cells and cell survival^1,4,5^. Our data showed that BAFF–BAFFR engagement triggered SFK activation, which further induced potassium depletion and ROS generation to promote NLRP3 inflammasome assembly in 2-h. Expanding inflammasome activity was observed after 8-h BAFF treatment connecting with the up-regulation of *NLRP3* and *pro-IL-1β* expression and the participation of cIAPs in caspase-1 processing. Moreover, the development of inflammasome activities is affected by crosstalk between BAFFR and BCR signals. This crosstalk could activate Lyn kinase, blunt Src activities, and ultimately prevent occurrence of cell pyroptosis. This observation may explain why transgenic mice with BAFF over-expression could develop autoimmunity^7,8^.

While BAFFR, TACI, and BCMA can all bind to BAFF, BAFFR appears to play the dominant role for B cell survival^1^. It does so by potently activating the non-classical NF-κB pathway, leading to up-regulation of Mcl1^43^ and Pim2 kinase^44^, as well as to cytoplasmic retention of protein kinase C^45^. Alternately, here we showed that BAFF ligation to BAFFR, not to TACI or BCMA, could activate inflammasomes in B cells and B cell death in a time-dependent and dose-dependent fashion.

BAFFR signaling potently activates the non-classical NF-κB2 pathway and weakly activates the classical NF-κB1 pathway in B cells^46,47^. BAFF induces SFK activation, which has been shown to promote B cell survival in vitro^9^. SFK affects P2X_7_R function by binding to the C-terminal region of Src tyrosine kinase^41^. In turn, K^+^ efflux^48^ and Ca^2+^ signaling^49^ result in mitochondria stress and ROS production. Cytosolic ROS from either the mitochondria or produced by NOX can stimulate additional ROS production by activating the redox-sensing Src family kinases^42,50^. Our present study showed that suppression of SFKs pharmacologically reduced ROS generation and K^+^ efflux, leading to down-regulation of inflammasome activation. Several NLR molecules, together with the adaptor protein ASC and procaspase-1, form molecular platforms that are activated by ROS and potassium efflux^16^. Simultaneously, cIAPs–TRAF2–TRAF3 complex detaches and degrades TRAF3 after activation of BAFFR^1^. cIAPs–TRAF2 promotes pro-caspase-1 processing through ubiquitination of pro-caspase-1^23^. That pro-caspase-1 processing appeared more significant after 8-h treatment with BAFF probably results from primary inflammasome activation followed by inflammasome components increase after transcriptional induction and cIAPs–TRAF2 modulation.

The crosstalk between BCR and BAFFR via activation of both NF-κB pathways suggests that their regulation of B cell survival is interconnected. Antigen binding to BCR initially leads to phosphorylation of ITAM in BCR-associated Igα-chain and Igβ-chain^51^. This initial phosphorylation of ITAM is mediated by SFKs, and is a prerequisite for Syk recruitment and activation^33^. Among SFKs, Lyn plays a prominent role to initiate BCR signaling, and functions to promote or inhibit immune cell activation depends on the types of the stimulus and the developmental state of B cells^11,34^. Our present study demonstrates BCR activation or Lyn activation by a specific stimulator that strongly attenuated BAFF-induced inflammasome activation in the three types of B cells tested.

In conclusion, in addition to previous studies that implicate the role of BAFF in B cell survival, our findings support the hypothesis that BAFF may trigger inflammation in B cells through inflammasome activation. We also observed a connection between BAFFR and BCR, whereby BCR activities suppressed BAFF-driven inflammasome activation and cell death. Further research on the interaction between BAFF–BAFFR and Ag–BCR in B cell homeostasis remains to be explored.

## Supplementary information

CDDIS-20-2285 Supplementary Figure Legends

Supplementary Figure S1

Supplementary Figure S2

Supplementary Figure S3

Supplementary Figure S4

Supplementary Figure S5
