# Supplementary material for: BAFF-driven NLRP3 inflammasome activation in B cells
Source: Cell Death Dis. 2020 Oct 1;11(9):820. doi: 10.1038/s41419-020-03035-2 (PMC7529748; doi:10.1038/s41419-020-03035-2)
Supplement: Supplementary file 1 — CDDIS-20-2285 Supplementary Figure Legends [file 41419_2020_3035_MOESM1_ESM.docx]

**Supplementary Information**

**BAFF-Driven NLRP3 Inflammasome Activation in B Cells**

Ken-Hong Lim,^1,2,3^ Lih-Chyang Chen,^2^ Kate Hsu^4,8^, Chia-Ching Chang,^5,8^ Chia-Yu Chang,^5^ Chen-Wei Kao,^3^ Yi-Fang Chang,^1,2,3^ Ming-Chih Chang,^1,2^ Caleb Gonshen Chen,^1,2,3,6,7^

**Table: Resources of Materials**

**Fig S1, Time-elapse expression of (A) *NLRC4*, (B) *NLRP1*, and (C) *NLRP3* in B cells after treatment with BAFF**

**Fig S2, Caspase-1 activity and IL-1β production of primary CD19+ isolated B cells**

**Fig S3, Increase in intracellular Ca^2+^ in BAFF-treated B cells**

**Fig S4, BAFF-directing caspase-1 activation is irrelevant to TACI and BCMA receptors**

**Fig S5, B cells with active Caspase-1 reacting to FLICA**

**Supplementary Table. Key Resources of Materials**

| Reagent and Resource | Source | Identifier |
| --- | --- | --- |
| Antibody | | |
| Human NLRP3 (aa 4-223) antibody | R&D | AF6789 |
| Human NLRP3 PE-conjugated antibody | R&D | IC7578P |
| Caspase-1 (D7F10) Rabbit mAb | Cell Signaling | 3866 |
| Cleaved Caspase-1 (Asp297) (D57A2) Rabbit mAb | Cell Signaling | 4199 |
| IL-1β antibody (C-20) | Santa Cruz | sc-1250 |
| cIAP1 antibody (F-4) | Santa Cruz | sc-271419 |
| cIAP2 antibody (Ac-71) | Santa Cruz | sc-517317 |
| TRAF2 antibody (H-10) | Santa Cruz | sc-7346 |
| TRAF3 antibody (G-6) | Santa Cruz | sc-6933 |
| Anti-ASC (TMS1) (Human) | MBL | D086-3 |
| Anti-Actin antibody | Merck Millipore | MAB1501 |
| Phospho-Src Family (Tyr416) antibody | Cell Signaling | 2101 |
| Phospho-Src (Tyr527) antibody | Cell Signaling | 2105 |
| Src (36D10) Rabbit mAb | Cell Signaling | 2109 |
| Human BAFF-R antibody | Q96RJ3**,** R&D | AF1162 |
| Lyn antibody | Cell Signaling | 2732 |
| Anti-Human IgG | SouthernBiotech | 2020-01 |
| Anti-Human IgM | SouthernBiotech | 2040-01 |
| Anti-Human IgD | SouthernBiotech | 9030-01 |
| Human TACI antibody | R&D | MAB174 |
| Human BCMA antibody | R&D | MAB193 |
| PerCP/Cy5.5 anti-mouse CD19 antibody | BioLegend | 152405 |
| Ovalbumin, Alexa Fluor 488 conjugate | Invitrogen | O34781 |
| APC/FITC Annexin V | BioLegend | 640920 |
| Human GSDMD rabbit polyclonal antibody | Sigma-Aldrich | G7422 |
| Chemicals, Peptides, and Recombinant Proteins | | |
| Human BAFF protein | R&D | 2149-BF |
| Mouse BAFF protein | R&D | 8876-BF |
| Protein G | Invitrogen | 1004D |
| Lipofectamine 2000 | Invitrogen | 11668019 |
| Disuccinimidyl Suberate (DSS) | Thermo Scientific | 21655 |
| RIPA Buffer | Thermo Scientific | 89900 |
| CM-H2DCFDA | Invitrogen | C6827 |
| Diphenyleneiodonium (DPI) | Sigma-Aldrich | D2926 |
| N-acetyl-L-Cysteine (NAC) | Sigma-Aldrich | A7250 |
| (2R, 4R)-APDC | Enzo Lifscience | ALX-550-178 |
| Potassium Chloride  Propidium iodide | Merck Millipore  BioLegend | 1.04936  421301 |
| PP1 | Cayman Chmical | 14244 |
| MLR 1023 | TOCRIS | 4582 |
| Fast SYBR Green Master Mix | Applied Biosystems | 4385612 |
| Prolong Gold Antifade Mountant with DAPI | Invitrogen | P36935 |
| Fluo-4 AM | Invitrogen | F14201 |
| Nitric acid | Merck Millipore | 100456 |
| Apyrase (oxATP) | Sigma | A6237 |
| Ovalbumin  zVAD-FMK  zYVAD-FMK  Puromycin | InvivoGen  Calbiochem  Calbiochem  Sigma-Aldrich | Vac-pova  Cas 187389-52-2  Cas 218746  58-58-2 |
| siRNA and shRNA | | |
| cIAP1 siRNA (h) | Thermo Fisher | 121289 |
| cIAP2 siRNA (h) | Thermo Fisher | 121285 |
| NLRP3 shRNA (h) | RNAi Core Facility | TRCN0000062723 |
| LYN shRNA (h) | RNAi Core Facility | TRCN00000218210 |
| LYN siRNA (h) | Santa Crusz | sc-29393 |
| Caspase-1 siRNA (h) | Santa Cruz | sc-29235 |
| Caspase-1 shRNA (h) | Santa Cruz | sc-156046V |
| Critical Commercial Assays | | |
| Caspase-1 Assay Kit (Fluorometric) | abcam | ab39412 |
| Human IL-1β ELISA Kit | R&D | DLB50 |
| Human CD19 Positive Selection Kit | StemCell Technologies | 18054 |
| High-Capacity DNA Transcription Kit | Applied Biosystems | 4374967 |
| Human B Cell Expansion Kit | R&D | CDK005 |
| in vitro DNA & siRNA Transfection Reagent | Polyplus | 712-60 |
| FAM-FLICA in vitro Caspase-1 Detection Kit | Immunochemistry Technologies | 98 |
| FITC Annexin V Apoptosis Detection Kit with PI | BioLegend | 640914 |
| Experimental Models: Cell Lines | | |
| JM1 | ATCC | 60513 |
| SU-DHL-4 | ATCC | CRL-2957 |
| Software and Algorithms |  |  |
| FCS express 6.0 | De Novo Software | N/A |
| Axio Vision | Olympus | N/A |
| GraphPad Prism 6 (Win) | [National Institutes of Health](https://zh.wikipedia.org/wiki/National_Institutes_of_Health) | N/A |

**Fig S1. Time-elapse expression of (A) *NLRC4*, (B) *NLRP1*, and (C) *NLRP3* in B cells after treatment with BAFF,** Related to Fig 1. Quantitative PCR (qPCR) was performed using the CFX Connect^TM^ Real-Time System and software (Bio-Rad). Products were detected using SYBR^®^ Green Master Mix. PCR primer sequences are listed as follows: *NLRC4* Forward 5′-TGC ATC ATT GAA GGG GAA TCT G-3′ Reverse 5′-GAT TGT GCC AGG TAT ATC CAG G-3′. *NLRP1* Forward 5′-ATT CCA GTT TGT GCG AAT CCA-3′ Reverse 5′-GTT CCT TGG GGA GTA TTT CCA G-3′. *NLRP3* Forward 5′-GAT CTT CGC TGC GAT CAA CAG-3′ Reverse 5′-CGT GCA TTA TCT GAA CCC CAC-3′. The human *RPL19* gene was used as an internal reference control for normalization. *p < 0.05, **p < 0.01 (Student’s t-test).

**Fig S2. Caspase 1 activity (A) and IL-1β production (C) of primary CD19+ isolated B cells compared with CD3+ isolated T cells as controls (B and D, respectively).** Related to Fig 1. Primary B cells from PBMC of 8 healthy donors were isolated using CD19+ positive selection and cultured in RPMI media for 3 days before treatment with BAFF for the indicated periods of time. Primary T cells were isolated using CD3+ positive selection for controls. Equivalent amount of cell lysates were assayed for their ability to cleave a fluorescent caspase-1 substrate, YVAD-AFC. Quantification of caspase-1 activity was determined using fluorometer assay. IL-1β levels of supernatants were measured using an enzyme linked immunosorbent assay (ELISA) kit from R&D Systems according to the manufacturer’s instructions. A caspase-1 specific inhibitor, zYVAD-FMK, was used to verify the production of IL-1β correlated with the interplay of BAFF and caspase-1 activation. AFU, arbitrary fluorescence units. *p < 0.05, **p < 0.01, ***p < 0.001 (Student’s *t*-test).

**Fig S3. Increase in intracellular Ca^2+^ in BAFF-treated B cells,** Related to Fig 4. Representative profiles of Ca^2+^ oscillation in (A, left panel) JM1, (B, left panel) SU-DHL-4, and (C, left panel) primary B cells treated with BAFF in the absence or presence of P2X_7_R antagonist oxATP (100 μM). The MFI of the fluo4–Ca^2+^-complex was analyzed at intervals of 20 s. Area under the curve (AUC) of intracellular Ca^2+^ from three independent experiments in (A, right panel) JM1, (B, right panel) SU-DHL-4, and (C, right panel) primary B cells. Error bars represent mean ± SEM. Asterisks indicate significant differences between treatment with BAFF and other treatments. *p < 0.05, **p < 0.01 (two-tailed, unpaired Student’s *t*-test).

**Fig S4. BAFF-directing caspase-1 activation is irrelevant to TACI and BCMA receptors,** Related to Fig 5. B cells that were previously preincubated with (A) anti-TACI antibodies or (B) anti-BCMA antibodies were treated with BAFF (200 ng/ml) for 24-h. Populations of FLICA+ B cells were determined by flow cytometry.

**Fig S5. B cells with active Caspase-1 reacting to FLICA,** Related to Fig 6. JM1, SU-DHL-4, and primary B cells were left untreated or treated with BAFF in the absence or presence of PP1 (10 μM). Green fluorescence represents Caspase-1 reaction with fam-FLICA.
