# Supplementary figures and images for: BAFF-driven NLRP3 inflammasome activation in B cells
Source: Cell Death Dis. 2020 Oct 1;11(9):820. doi: 10.1038/s41419-020-03035-2 (PMC7529748; doi:10.1038/s41419-020-03035-2)

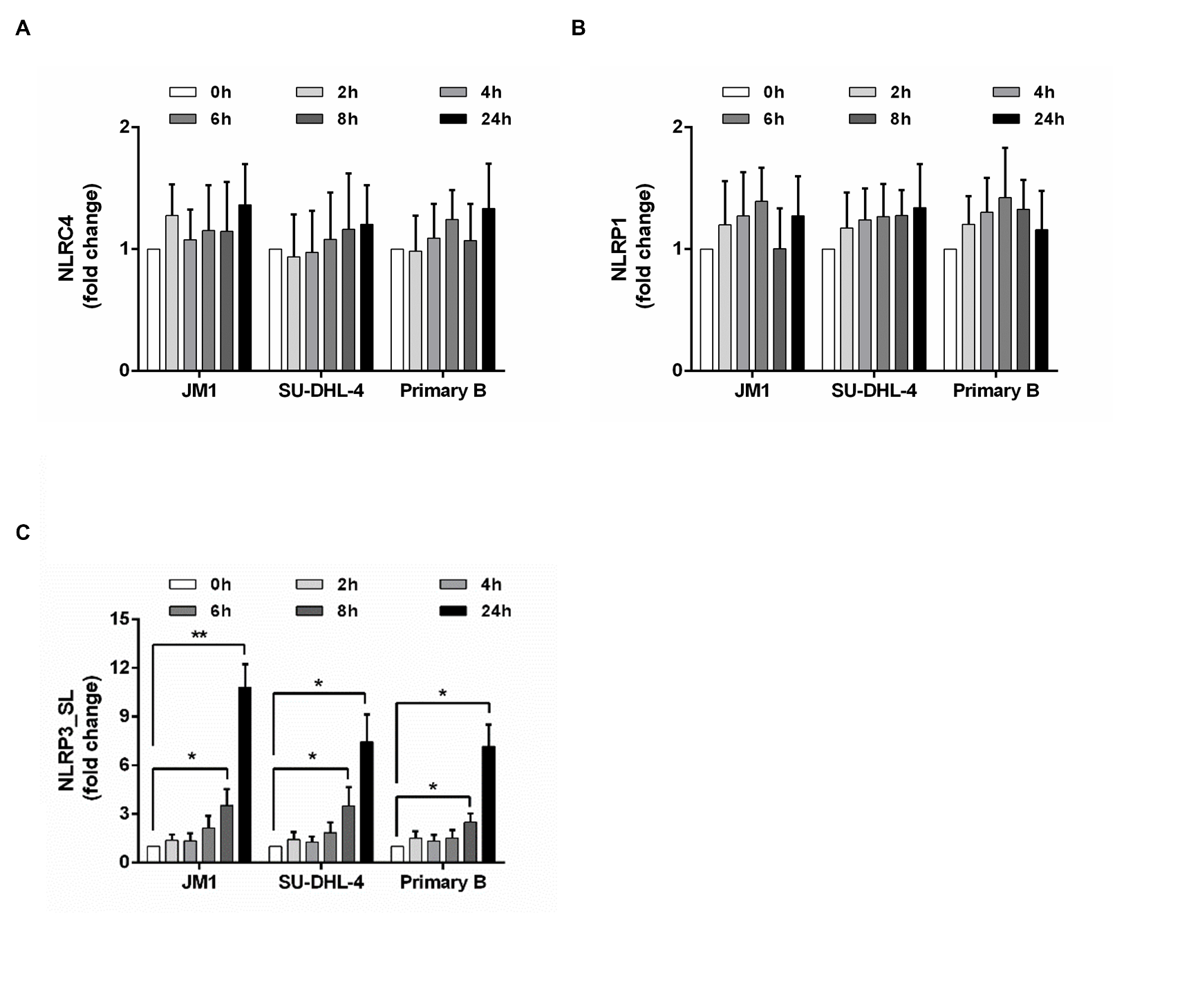

Supplement: Supplementary file 2 — Supplementary Figure S1 [file 41419_2020_3035_MOESM2_ESM.tif]

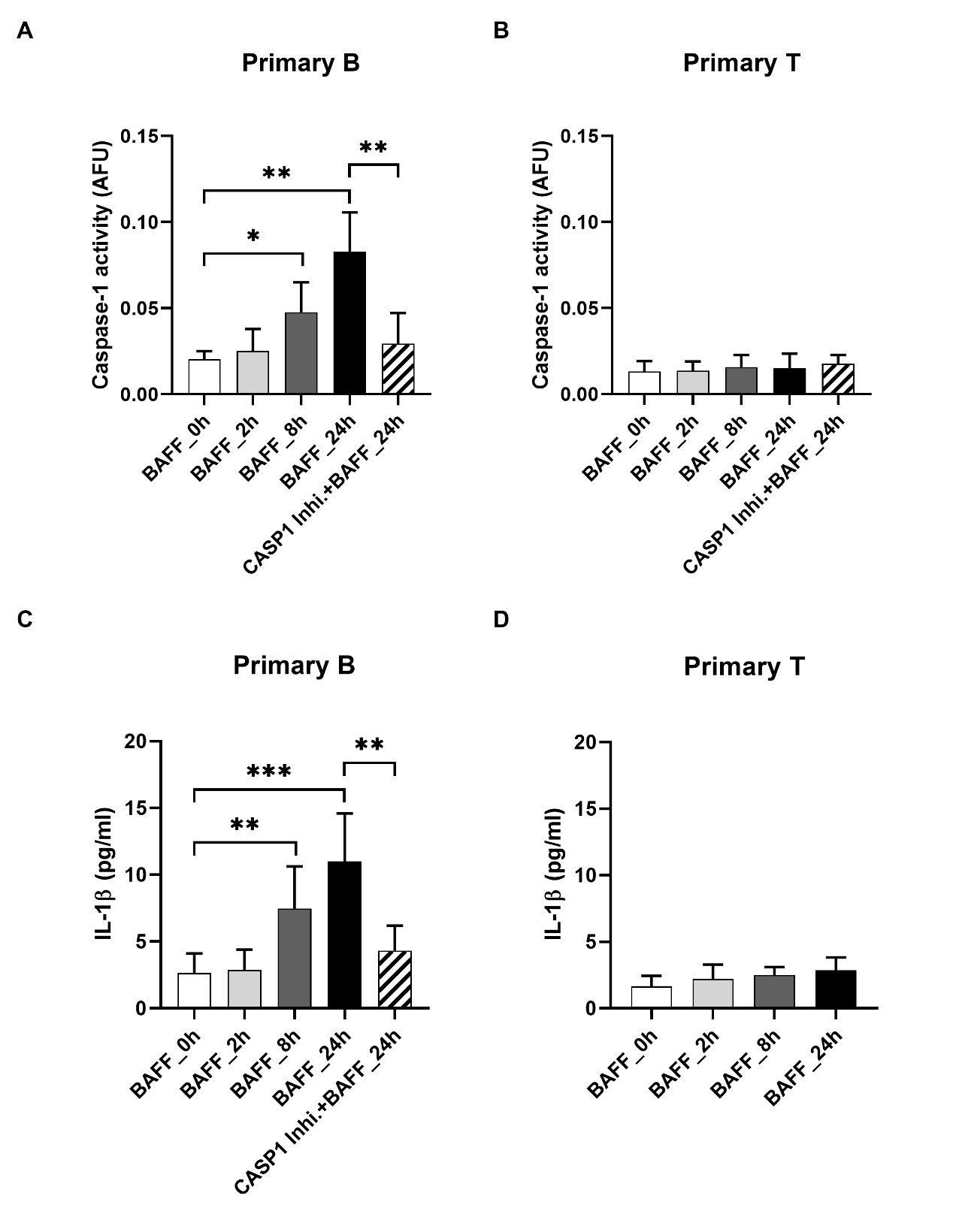

Supplement: Supplementary file 3 — Supplementary Figure S2 [file 41419_2020_3035_MOESM3_ESM.tif]

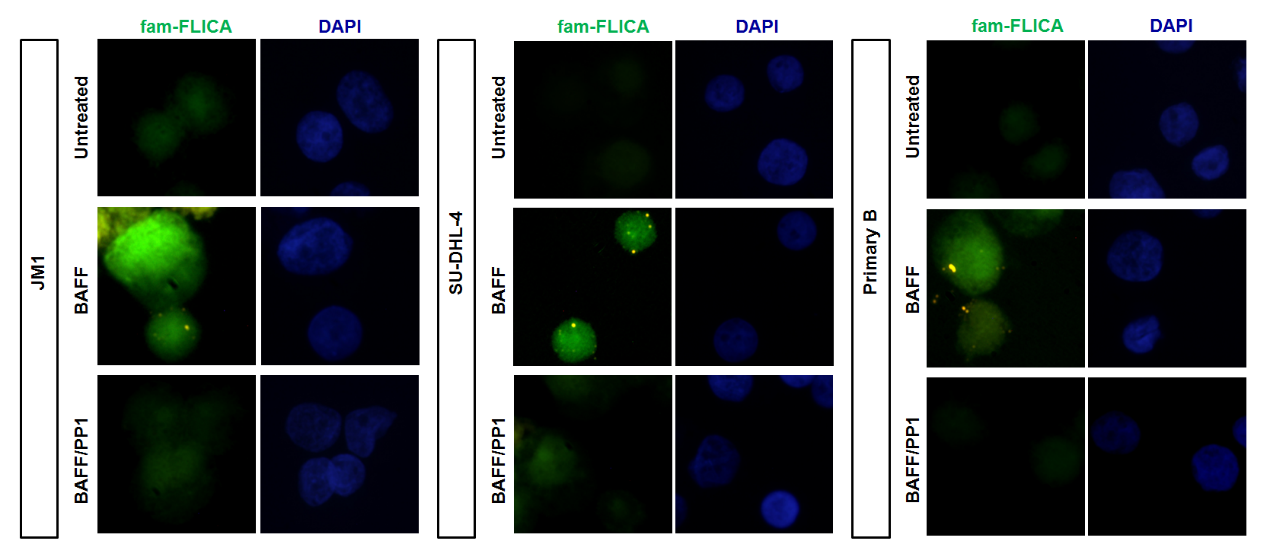

Supplement: Supplementary file 6 — Supplementary Figure S5 [file 41419_2020_3035_MOESM6_ESM.tif]
